# Abortion in Cows

**Published:** 1883-04

**Authors:** 


Abortion in Cows.—We should be glad if some of our readers would again attack the subject of abortion in cows, which is still the reproach of veterinary science. Neither prizes nor commissions, nor learned societies have yet discovered a cause or cure for it. Of late it has been appearing almost epidemically among some of the valuable Jersey herds in our neighborhood. Dr. Ehrick Parmly, of Seabright, N. J., is reported to have lost nine valuable calves by "slipping," within a short time. The same trouble has threatened the Hilltop herd of Mr. G. W. Farlee, of Trenton, who owns probably the finest bred lot of Jerseys in the country. The best remedy for threatened abortion, and one which has been efficacious in the herds mentioned, is half ounce doses of asafœtida. As there is unquestionably a nervous element in the disease this remedy is a rational one.

